# CIAPIN1 nuclear accumulation predicts poor clinical outcome in epithelial ovarian cancer

**DOI:** 10.1186/1477-7819-10-112

**Published:** 2012-06-19

**Authors:** Xiaolan Cai, Jian Wang, Xiaoyan Xin

**Affiliations:** 1 Department of Gynecology and Obstetrics, Xijing Hospital, the Fourth Military Medical University, Number 15, Changle Western Road, Xi’an, 370032, China; 2 The Second sanatorium of Jinan Military area, Number 1, Taipinjiao 6 Road, Qingdao, 266071, China

**Keywords:** CIAPIN1, Epithelial ovarian cancer, Prognosis, Nuclear localization

## Abstract

**Background:**

Epithelial ovarian cancer (EOC) is an aggressive disease with poor prognosis. The expression of cytokine-induced apoptosis inhibitor 1 (CIAPIN1) correlates with the malignant progression of several cancers. However, the relationship between the subcellular localization of CIAPIN1 and clinical characteristics in EOC remains unclear.

**Methods:**

Immunohistochemistry was performed to detect CIAPIN1 expression in 108 EOC tissues. CIAPIN1 expressions in eight fresh EOC tissues were detected by Western blotting. The relationship between CIAPIN1 subcellular expression and patients’ clinicopathological features, including prognosis, was evaluated. Immunohistochemistry and immunofluorescence were employed to assess the CIAPIN1 subcellular localization in the EOC cell lines A2780 and HO8910. In addition, all patients were followed up to assess the prognostic value of CIAPIN1 in patients with EOC.

**Results:**

CIAPIN1 is highly expressed in EOC, but is present at low levels in paired non-cancerous ovarian epithelial tissues. The results of Western blotting were in accordance with the immunohistochemical results. Poor differentiation of the tumors and EOC cell lines correlated with higher levels of CIAPIN1 nuclear expression. CIAPIN1 nuclear expression significantly correlated with the Federation International of Gynecology and Obstetrics (FIGO) stage and histological differentiation (*P* = 0.034 and *P* < 0.0001, respectively). Moreover, nuclear localization of CIAPIN1 was selected as an unfavorable prognostic factor by both univariate and multivariate analyses ( *P* < 0.001). However, no significant correlations were observed between cytoplasmic localization of CIAPIN1 and clinicopathological parameters.

**Conclusions:**

CIAPIN1 might play a crucial role in the differentiation of EOC cells. Elevated expression of nuclear CIAPIN1 negatively correlated with the survival of EOC patients, suggesting that nuclear CIAPIN1 might serve as a prognostic biomarker for EOC patients.

## Background

EOC is the fourth leading cause of cancer-related deaths among women worldwide [1,2]. Despite considerable advances in surgical techniques and neoadjuvant chemotherapy, overall patient treatment outcomes have not substantially improved [3]. EOC remains a clinical challenge, primarily because of the absence of effective methods for early diagnosis and lack of prognostic markers. Therefore, additional efforts are required to identify biomarkers for predicting poor outcome in EOC.

CIAPIN1, a novel anti-apoptotic molecule, has been identified to be a downstream effector of the receptor tyrosine kinase-Ras signaling pathway in the mouse Ba/F3 pro-B cell line [4]. CIAPIN1 has been demonstrated to be ubiquitously distributed in normal fetal and tumor tissues, with high expression in actively metabolic tissues [5,6]. Therefore, CIAPIN1 is likely involved in important physiological functions in tumors. Additionally, a previous study has shown that CIAPIN1 is localized not only in the cytoplasm, but also in the nucleus, and the authors suggest that subcellular localization of CIAPIN1 might be significant for its function [7]. Moreover, CIAPIN1 has been demonstrated to be a critical molecule involved in tumor aggressiveness and may represent a prognostic marker for patient outcome in several types of cancer, such as hepatocellular cancer [8] and leukemia [9]. In recent studies, the significance of CIAPIN1 has been identified in tumors such as esophageal cancer and colorectal cancer; however, the correlations were detected between the expression level of CIAPIN1 in both the nucleus and cytoplasm, and tumor clinicopathological features. CIAPIN1 subcellular localization has not been examined in tumors. Therefore, improved knowledge of the subcellular localization of CIAPIN1 in tumors will be instrumental for the design of optimal strategies to selectively disrupt CIAPIN1 in human cancers.

The clinical significance of CIAPIN1 subcellular localization has not yet been examined in EOC. In this context, this study evaluated the expression of CIAPIN1 in EOC tissues and analyzed the clinical significance of the subcellular localization of CIAPIN1. Our data revealed that CIAPIN1 nuclear localization might provide additional information regarding EOC patient outcome, and represents a valuable biomarker for diagnosis and postoperative predictions of EOC.

## Methods

A total of 108 EOC tissues and paired non-cancerous tissues from primary EOC patients were surgically obtained between 1998 and 2006 in Xijing Hospital, the Fourth Military Medical University, Xi’an, China. The median patient age was 53 years (range: 32–68 years). All patients agreed to the procedure and signed consent forms. This study was authorized by the Hospital’s Protection of Human Subjects Committee. No patient had received chemotherapy or radiation therapy prior to surgery. All of the patients died of EOC.

The tissue specimens were obtained from surgery, formalin-fixed and paraffin-embedded. The paired non-cancerous tissues were epithelial tissues from ovarian biopsies or ovarian surface scrapings. Each sample was cut in 4-μm sections, and one section was stained with hematoxylin-eosin and used for morphological diagnosis. Patients’ characteristics, such as age, FIGO stage, histological differentiation, lymph node status and pathology subtype, were obtained from the medical records. The patient characteristics are summarized in Table 1. The diagnosis of EOC was confirmed by histological analysis by three pathologists. The survival information from the postoperative follow-up of all 108 patients was received by telephone or mail. The median follow-up time was 34 months (range: 6–86 months). An additional eight intraoperative fresh EOC tissues and paired non-cancerous ovarian epithelial tissues were excised, and immediately stored in liquid nitrogen for Western blotting analysis.

**Table 1:** **Correlation between CIAPIN1 nuclear expression and clinicopathological parameters (*P*** **< 0.05 statistically significant)**

**Parameters**	**Total**	**Nucleus expression**				
		−	+	++	+++	*p*-value
Age						0.748
<53	59	7	16	23	13	
≥53	49	3	16	19	11	
Pathology subtypes						0.517
Serous cyst	45	3	16	15	11	
Other types	63	7	16	27	13	
FIGO stage						0.034
I + II	55	6	20	23	6	
III + IV	53	4	12	19	18	
Histological differentiation						<0.0001
Well	33	7	11	15	0	
Moderately	33	2	5	21	5	
Poorly	42	1	16	6	19	
Lymph node status						0.523
Positive	27	4	9	10	4	
Negative	81	6	23	32	20	

### Immunohistochemistry (IHC)

Immunohistochemistry (IHC) staining was performed as previously described [10,11]. The primary mouse monoclonal anti-CIAPIN1 antibody (dilution 1:800) was developed in our laboratory [12]. Mouse anti-immunoglobulin G (Pierce, Rockford, IL, USA) was used as the negative control. An anti-APE1 mouse monoclonal antibody (1:5,000; Novus Biological, Littleton, CO) was used as the positive control. Each tissue specimen was de-waxed twice with xylene and gradually hydrated. After using a pressure cooker with 10 nM citrate buffer (PH 6.0) for 5 min, the immunostaining procedure was performed. Endogenous peroxidases were blocked with 3% H_2_O_2_-methanol for 10 min, and the samples were incubated with the primary monoclonal antibody overnight at 4 °C, rinsed three times for 5 min in phosphate-buffered saline (PBS) and incubated with a horseradish peroxidase-conjugated anti-IgG antibody (1:4,000; Santa Cruz) for 1 h. Finally, the sections were developed with diaminobenzidine solution for 2 min, washed briefly in running water, counterstained with hematoxylin, dehydrated through a graded series of alcohol to xylene and coverslipped. Three pathologists who did not know the clinical features or survival status of the patients then viewed the stained tissue slides separately.

To clarify the association between CIAPIN1 subcellular localization and clinicopathological characteristics, the nuclear and cytoplasmic expression of CIAPIN1 was examined and evaluated separately. An immunoreactivity score (IRS) system was applied using the following standards: (1) The nuclear expression of CIAPIN1 was scored by estimating the proportion of tumor cells with positive nuclear staining (0, none; 1, ≤10%; 2, 10 to ≤ 25%; 3, 26 to ≤ 50%; and 4, >50%). The proportion score was based on the proportion of tumor cells with positive cytoplasmic staining (0, none; 1, ≤10%; 2, 10 to ≤ 25%; 3, 26 to ≤ 50%; and 4, >50%). (2) The intensity score was assigned for the average intensity of positive tumor cells (colorless scored 0, pallid scored 1, yellow scored 2, and brown scored 3). The nuclear or cytoplasmic score for CIAPIN1 was the product of the proportion and intensity scores, ranging from 0 to 12. The immunoreactivity scoring was ranked as absent (−, score 0), weak (+, score 1–4), moderate (++, score 5–8) or strong (+++, score 9–12) according to the proportion and staining intensity. Images were obtained under a light microscope (Olympus BX51, Tokyo, Japan) equipped with a DP70 digital camera.

### Western blotting analysis

Eight intraoperative fresh EOC specimens and paired non-cancerous ovarian epithelial specimens were sonicated with an ultrasonic tissue disrupter in lysis buffer for 30 min. The tissue debris was pelleted by centrifugation, and supernatants were collected. The total protein (30 ug was separated by 12% SDS-polyacrylamide gel electrophoresis for 45 min and electroblotted onto a nitrocellulose membrane for 30 min. Non-specific binding was blocked with 5% non-fat milk in TBS for 1 h at room temperature. Then, the membrane was hybridized using the primary antibodies Mab CIAPIN1 (1:2,000) or β-actin (1:4,000, Sigma) overnight at 4 °C. After three washes with TBS-T, the NC membrane was incubated at room temperature for 1.5 h with HRP-conjugated goat anti-mouse antibodies. The membrane was rinsed three times with TBS-T for 25 min, and specific protein bands were visualized using ECL (Santa Cruz Biotechnology, Inc.) and a Bio-Rad (Thermo, USA) system.

### Immunofluorescence (IF)

The A2780 cell line was originally derived from a female ovarian adenocarcinoma, and the HO8910 cell line was originally derived from a female ovarian serous carcinoma. These two cell lines were preserved in our laboratory. Cells were seeded on glass cover slips in six-well plates and cultured in high-glucose Dulbecco’s modified Eagle's medium (HyClone, Thermo, UT) supplemented with 10% (V/V) fetal calf serum (FCS, Sigma), 100 IU/ml penicillin and 100 mg/ml streptomycin at 37 °C in a humidified chamber with 5% CO_2_. Afterward, the cells were fixed by incubation in 4% paraformaldehyde for 5 min and permeabilized in PBS containing 0.1% Triton X-100 for 10 min. Non-specific binding was blocked with 10% BSA for 30 min. CIAPIN1 was stained using a 1:800 dilution of the antibody in 1% BSA overnight at 4 °C. A mouse FITC-conjugated phalloidin secondary antibody (Invitrogen) was then used at a 1:1,000 dilution and incubated for 2 h in a humidified chamber with minimal exposure to light. All washes were performed in 1× PBS. An anti-fade solution containing DAPI (Vector Laboratories, CA) was used in mounting the slides. Images were taken with a fluorescence microscope (Olympus BX51, Tokyo, Japan), and Adobe Photoshop was used to merge the images.

### Statistical analysis

Nominal variables were compared using the *χ*^2^ test, and ordinal categorical variables were evaluated by a non-parametric Spearman’s rank test. The survival probabilities of patients were described by Kaplan-Meier curves and compared using the log-rank test. The Cox proportional hazards model with likelihood ratio statistics was employed to further evaluate the risk factors for survival. Two-tailed *P* < 0.05 was considered to be statistically significant. The SPSS18.0 software package (SPSS, Inc., Chicago, IL) was used for the statistical analyses.

## Results

### Elevated expression of CIAPIN1 in EOC

**Figure 1:**
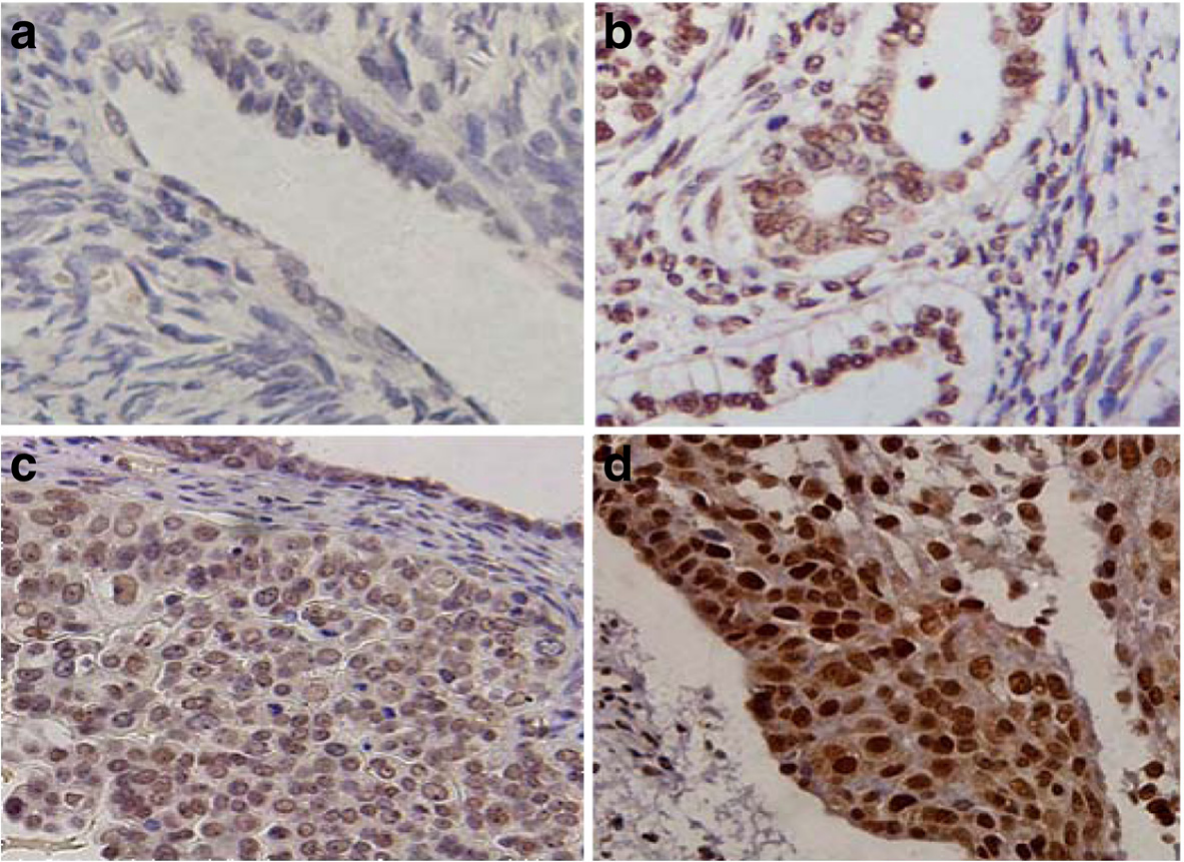
**Expression of CIAPIN1 in EOC tissues and paired non-cancerous tissues by immunohistochemistry**. ( **a**) Negative staining (−) of CIAPIN1 in paired non-cancerous tissues. ( **b**) Positive staining (+) of CIAPIN1 in the nucleus in well-differentiated tumor tissue. ( **c**) Moderate nuclear positive staining (++) of CIAPIN1 in moderately differentiated EOC tissues. ( **d**) Strong nuclear staining (+++) of CIAPIN1 in poorly differentiated EOC tissues. (Original magnification, 200×).

CIAPIN1 expression was investigated by IHC in 108 EOC tissues and paired non-cancerous tissues. Positive CIAPIN1 staining was ubiquitously observed in both the cytoplasm and the nucleus of EOC cells (Figure 1). In the paired non-cancerous tissues, negative expression of CIAPIN1 was detected in 45.3% (49/108) of the specimens. In contrast, negative nuclear expression of CIAPIN1 was observed in 9.3% (10/108) of the specimens, and negative cytoplasmic expression of CIAPIN1 was observed in 37.0% (40/108) of the cases. CIAPIN1 nuclear expression was significantly elevated in EOC compared with paired non-cancerous samples (*P* < 0.001), but CIAPIN1 cytoplasmic expression in EOC was not significantly different from that in paired non-cancerous tissues ( *P* > 0.05). In the ovarian cancer tissues, positive nuclear immunoreactivity was observed in 98 (90.7%) specimens, with 32 (30%) displaying weak (+) positive expression, 42 (39%) moderate (++) positive expression and 24 (22%) strong (+++) positive expression. In contrast, positive nuclear immunoreactivity was observed in 68 (37%) samples, with 30 (28%) displaying weak (+) positive expression, 22 (20%) moderate (++) positive expression and 16 (15%) strong (+++) positive expression.

### Quantitative analysis of CIAPIN1 expression in clinical specimens

**Figure 2:**
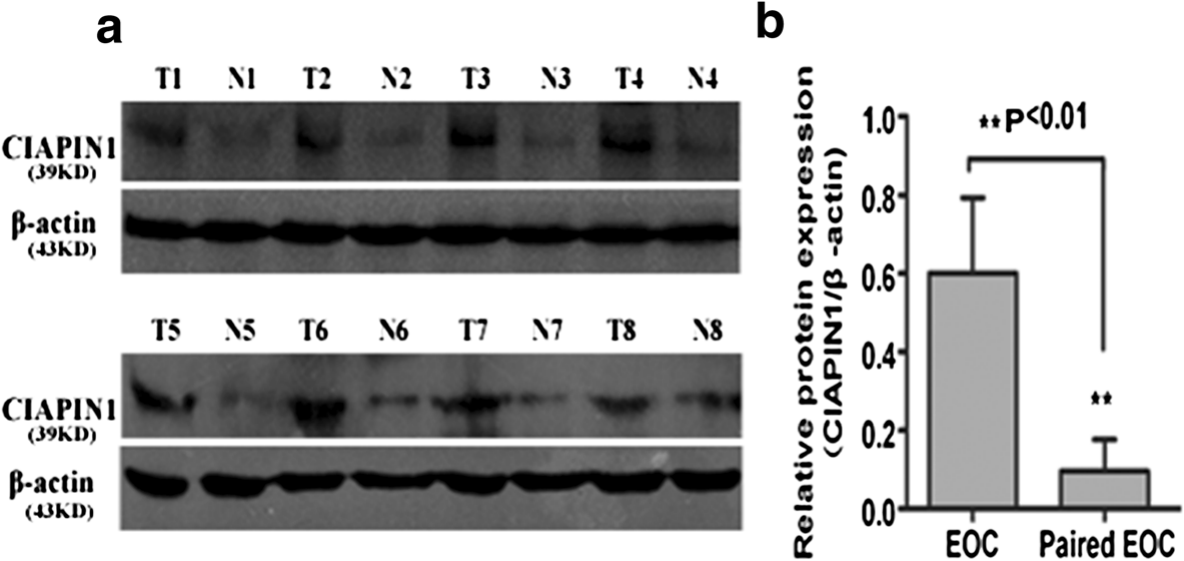
**CIAPIN1 expression in EOC and paired non-cancerous epithelial tissues by western blotting.** ( **A**) Eight representatives are shown. *T*, tumor tissue; *N*, paired non-cancerous epithelial tissue. β-actin was used as an internal control. ( **B**) Analysis of the CIAPIN1 protein expression in EOCs. CIAPIN1 had high expression in EOC, but low or no expression in paired non-cancerous ovarian epithelial tissues ( *Paired EOC*). (Statistical significance *P* < 0.01).

To confirm the immunohistochemical results, we further performed Western blotting on ovarian cancer specimens and paired non-cancerous ovarian epithelial specimens. As expected, the results supported the immunohistochemical results. Quantitative analysis of Western blotting analysis showed that CIAPIN1 expression was clearly elevated in the eight EOC tissues compared with the paired non-cancerous ovarian epithelial tissues (Figure 2A). The result showed that expression of CIAPIN1 protein was significantly higher than that in paired non-cancerous ovarian epithelial specimens (Figure 2B, *P* < 0.01).

### Correlation between the subcellular localization of CIAPIN1 and clinicopathological features

The correlation between the subcellular localization of CIAPIN1 and clinicopathological features in EOC was further evaluated. The nuclear expression level of CIAPIN1 increased from well-differentiated to poorly differentiated EOC tissues (Figure 1b-d). In contrast, the level of CIAPIN1 cytoplasmic expression did not obviously differ between non-cancerous epithelial tissues and well or moderately differentiated tumor tissues (Figure 1a-c). As shown in Table 1, positive nuclear expression of CIAPIN1 positively correlated with the degree of differentiation, indicating that the CIAPIN1 nuclear expression in poorly differentiated cancer was significantly higher than that in well-differentiated cancer (*P* < 0.0001). Moreover, the nuclear expression was also significantly higher in patients with an advanced FIGO stage (III + IV) than in those with an early FIGO stage (I + II) ( *P* = 0.034). No significant association was observed between CIAPIN1 nuclear expression and patients’ age, lymph node metastasis or pathology subtype ( *P* > 0.05). In addition, no statistically significant correlation between CIAPIN1 cytoplasmic expression and clinicopathological features was observed (see Additional file 1: Table S1, *P* > 0.05).

### Relationship between CIAPIN1 subcellular expression and differentiation in EOC cell lines

**Figure 3:**
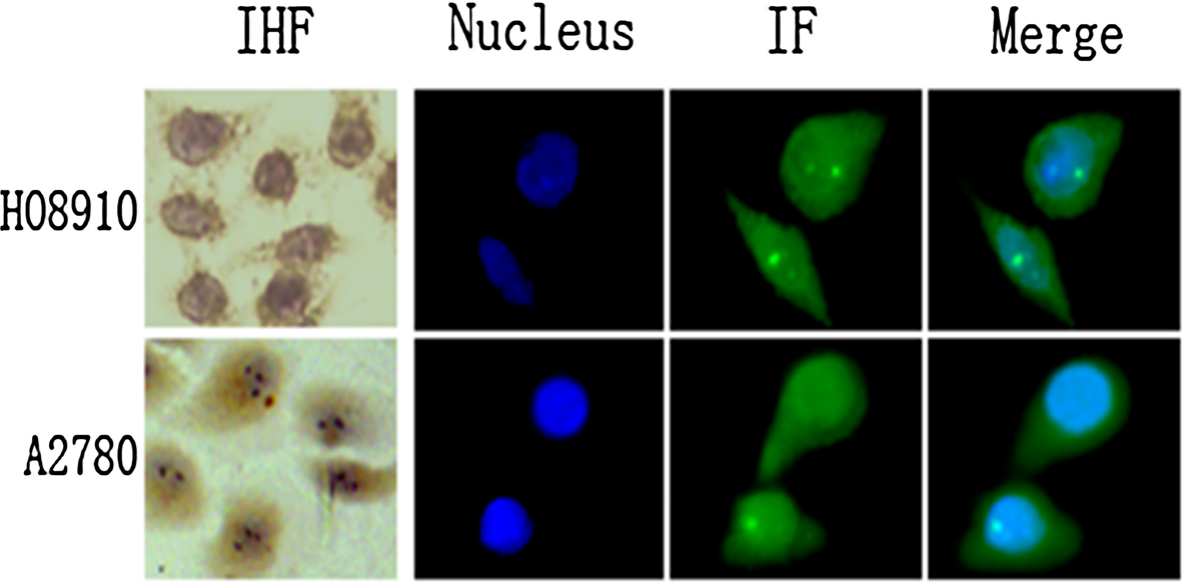
**Subcellular localization of CIAPIN1 in EOC cells. Morphology analysis of CIAPIN1 in EOC cell lines by immunohistochemistry (IHC) and immunofluorescence (IF).** High nuclear intensity of CIAPIN1 expression in A2780 cell lines and low nuclear intensity of CIAPIN1 in HO8910 cell lines.

The immunohistochemical results revealed that the nuclear expression of CIAPIN1 significantly increased from well-differentiated to poorly differentiated EOC. Because the A2780 cell line might be more poorly differentiated than the HO8910 cell line [13], the subcellular distribution of CIAPIN1 was also assessed in these cells. Immunofluorescence and immunohistochemical analyses (Figure 3) demonstrated that the nuclear immunoreactivity of CIAPIN1 was strong in A2780 cells and weak in HO8910 cells. However, no obvious difference in cytoplasmic immunoreactivity was observed in these cells.

### Survival analysis

**Figure 4:**
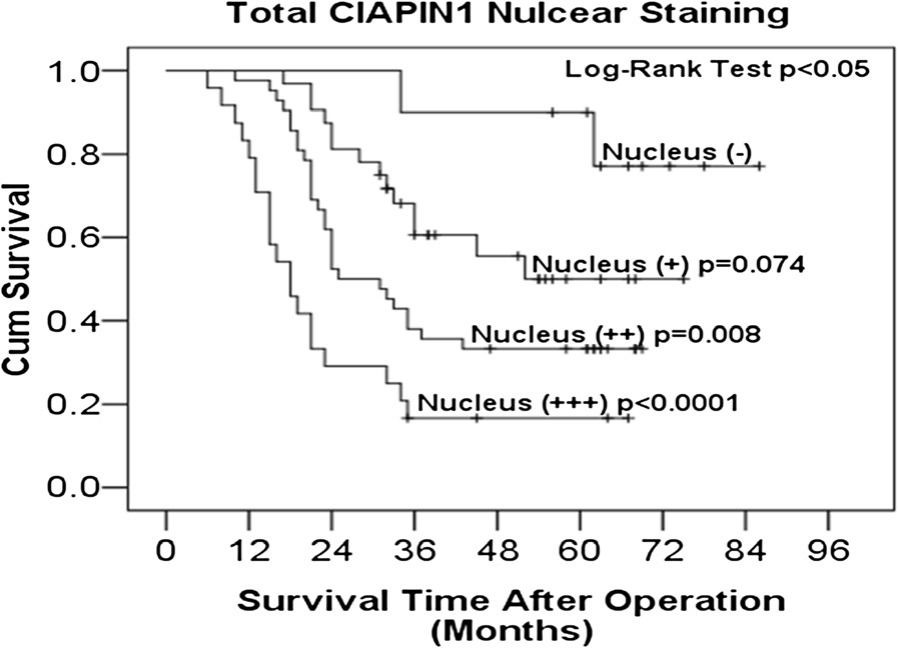
**Kaplan-Meier curves of postoperative survival of patients with nuclear localization of CIAPIN1 expression**. Patients with high nuclear expression of CIAPIN1 in EOC; the survival tended to a worse outcome compared to weak expression (log-rank test *P* < 0.05).

In univariate analysis (Table 2), CIAPIN1-positive nuclear expression, histological differentiation and FIGO stage were significantly associated with patient survival. Multivariate Cox proportional hazards regression analysis (Table 3) indicated that nuclear expression of CIAPIN1 was an independent predictive factor for poor overall survival in EOC patients (HR = 2.207, *P* < 0.0001). CIAPIN1 nuclear expression was then used to predict the postoperative survival rate for EOC patients by Kaplan-Meier analysis (Figure 4). Log rank analysis showed that the significance in weak (+), moderate (++) and poor (+++) CIAPIN1 nuclear localization staining was 0.074, 0.008 and <0.0001 compared with the negative staining group, respectively; however, no statistically significant difference was observed in the cytoplasmic expression of CIAPIN1 (see Additional file 1: Figure S1, *P* > 0.05). The 5-year survival rate of patients with negative (−) nuclear expression of CIAPIN1 was 90%, while the survival rate of patients with weakly positive (+), moderately positive (++) and strongly positive (+++) expression of CIAPIN1 was 50%, 33% and 16%, respectively.

**Table 2:** Univariate analysis of the association between clinical characteristics and survival in patients with EOC

**Clinicopathological features**	**Median survival time (months)**	**Kaplan-Meier P- value**
Age		0.295
<53	43	
≥53	31	
Pathology subtypes		0.759
Serous cyst	33	
Other types	34	
FIGO stage		0.004
I + II	52	
III + IV	28	
Histological differentiation		0.005
Well	35	
Moderately	33	
Poorly	22	
Lymph node status		0.703
Positive	33	
Negative	34	
Nucleus localization		<0.0001
−	-	
+	52	
++	25	
+++	18	

**Table 3:** Cox multivariate analysis

**Variable**	**Hazard ratio**	**95% confidence interval**	**P-value**
FIGO stage	1.598	0.959–2.665	0.072
Histological differentiation	0.978	0.708–1.350	0.892
Nucleus localization	2.003	1.443–2.781	<0.001

## Discussion

CIAPIN1 has been reported to be involved in proliferation [8], multidrug resistance [14], angiopoiesis [6] and anti-apoptosis [4,15] in many solid tumors and hematological malignancies. The intracellular distribution of CIAPIN1 has been demonstrated to play a cell death-defying role in a variety of mouse and human embryonic cells [4,5,7,9]. However, the subcellular distribution of CIAPIN1 in tumors and the relationship between CIAPIN1 expression and EOC had not been examined. The present study indicated that CIAPIN1 nuclear localization is associated with the histological differentiation, FIGO stage and prognosis for EOC.

This study demonstrated that CIAPIN1 is highly expressed in EOC tissues compared with paired non-cancerous epithelial tissues by immunohistochemistry and Western blotting. Consistently, CIAPIN1 has also been found to be elevated in hepatocellular carcinoma [8] and diffuse large B cell lymphoma [15]. These data indicate that CIAPIN1 may play an oncogenic role in diverse cancers. More importantly, this study revealed that high nuclear expression of CIAPIN1 is significantly associated with poor histological differentiation and an advanced FIGO stage in EOC. However, no correlation between CIAPIN1 cytoplasmic localization and clinical pathological features was observed in the EOC tissues. Interestingly, these results were in accordance with the observation that the levels of CIAPIN1 nuclear expression in A2780 cells were higher than those in HO8910 cells. The differentiation status of the A2780 cell line has previously been shown to be poorer than that of HO8910 [13]. Moreover, one expression pattern might be strictly regulated by the subcellular localization of various proteins and varied with alterations in function proteins, such as the subcellular localization of LIMK1, cathepsin L and ALCAM altered the prognosis of correlated cancers [16-18]. Thus, the study findings suggested that the nuclear expression of CAIPIN1 might be associated with the development of poorly differentiated EOC. Proper transport of a protein to its final destination is known to be crucial for the function of the protein [19]. The data from this study suggest that CIAPIN1 nuclear localization is also associated with carcinogenesis and differentiation in EOC, and at least partially with poor clinical outcome of EOC patients. These associations may be a result of the distinct differentiation status of the EOC cells or interactions with specific molecules. Further studies would be necessary to address this notion. From these data, we conclude that the nuclear distribution of CIAPIN1, but not the cytoplasmic distribution, plays an important role in EOC progression. We hypothesize that nuclear CIAPIN1 expression may be involved in neoplastic alterations, while the function of the cytoplasmic protein does not contribute to cancer progression.

This study demonstrated that CIAPIN1 expression has prognostic value in several human tumors [8,9,15] and revealed that patients with high levels of CAIPIN1 nuclear expression displayed shorter postoperative survival times than those with weak nuclear expression. This finding is in accord with previous studies that have indicated that subcellular localization is associated with poor prognosis in other tumors [17,18]. The studies suggest that nuclear expression of CIAPIN1 is closely related to the poor prognosis of patients with EOC. Moreover, multivariate Cox proportional hazards regression analysis revealed that CIAPIN1 nuclear localization is an independent poor prognostic factor in EOC. These data demonstrate that CIAPIN1 nuclear localization is a promising prognostic marker associated with shorter overall survival in EOC. This information might be useful for clinicians in providing individualized therapy for EOC patients with optimal benefit. However, the FIGO stage, pathological subtype and lymph node status were poor prognostic factors according to Cox multivariate analysis. These variables may be potential predictive factors for poor overall survival in EOC patients because of the potential correlations between clinicopathological variables and CIAPIN1 nuclear localization. Moreover, the differences may be caused by the cellular components among the ovarian specimens. Further studies of additional patient samples are necessary to address the significance of such correlations.

Bioinformatics prediction (PROSITE) analysis has indicated that CIAPIN1 is primarily localized in the nucleus and, to a lesser extent, in the cytoplasm [20]. The nuclear localization of CIAPIN1 is established either through specific nuclear localization signal sequences or via interaction with specific molecules. In fact, during mitosis, CIAPIN1 cooperates with posttranslational modifications of cell-cycle-dependent proteins, including cyclinD1 [21,22]. CyclinD1 can accelerate ovarian cancer carcinogenesis [23-26]. Thus, we hypothesize that CIAPIN1 in the nucleus might induce the development of EOC. In addition, the interaction of CIAPIN1 with PICOT (PKCθ interacting cousin of thioredoxin) at the N-terminal regions might lead to the growth of Ba/F3 cells [27]. Moreover, phosphorylated PKC can activate proliferation [28] and differentiation [29] through various signaling pathways. Recent studies have demonstrated that the stability and anti-apoptotic function of CIAPIN1 are the result of interactions with TXNL2 and PICOT [20]. TXNL2 is involved in the activation of the c-Jun N-terminal kinase/AP-1 and NF-kB pathways. These data imply that molecules in the cytoplasm that interact with CIAPIN1 might play crucial roles in causing the translocation of CIAPIN1 into the nucleus.

## Conclusions

To our knowledge, this study is the first to associate the subcellular expression of CIAPIN1 in EOC tissues with clinical parameters and patient outcomes using immunohistochemical methods. The results suggest that CIAPIN1 nuclear accumulation might be responsible for EOC progression and is likely associated with the neoplastic outcomes of EOC. The nuclear localization of CIAPIN1 may represent a useful diagnostic and prognostic marker for EOC.

## Competing interests

The authors declare that they have no competing interests.

## Authors’ contributions

XC performed the experiment and drafted the manuscript. JW participated in the data collection. XX acted as corresponding author and did the revisions. All authors read and approved the final manuscript.

## Supplementary Material

Additional file 1 **Table S1.** Correlation between CIAPIN1 cytoplasm expression and clinical pathological parameters ( *P* < 0.05 statistically significant). **Figure S1.** Kaplan-Meier curves of postoperative survival of patients with cytoplasmic localization of CIAPIN1 expression. Patients with high cytoplasmic expression of CIAPIN1 in EOC; the survival was not statistically significant (log-rank test, *P* > 0.05). Click here for file
