# Supplementary material for: CIAPIN1 nuclear accumulation predicts poor clinical outcome in epithelial ovarian cancer
Source: World J Surg Oncol. 2012 Jun 19;10:112. doi: 10.1186/1477-7819-10-112 (PMC3502349; doi:10.1186/1477-7819-10-112)

| Additional file 1 Correlation between CIAPIN1 cytoplasm expression and clinic pathological parameters (P < 0.05 statistically significant) | | |
| --- | --- | --- |
| Parameters | Total | Cytoplasm expression  - + ++ +++ P-value |
| Age  <53  ≥53 | 59  49 | 0.261  20 14 16 9  20 16 6 7 |
| Pathology subtypes  Serous cyst  Other types | 45  63 | 0.956  16 13 10 6  24 17 12 10 |
| FIGO stage  Ⅰ+Ⅱ  Ⅲ+Ⅳ | 55  53 | 0.391  19 13 12 11  21 17 10 5 |
| Histologic differentiation  Well  Moderately  Poorly | 33  33  42 | 0.071  20 4 5 4  7 11 9 6  13 15 8 6 |
| Lymph node status  Negative  Positive | 81  27 | 0.774  28 23 18 12  12 7 4 4 |
|  |  |  |

Additional file 2 Kaplan–Meier curves of postoperative survival of patients with cytoplasmic localization of CIAPIN1 expression. Patients with high cytoplasmic expression of CIAPIN1 in EOC, the survival was not statistically significant. (log-rank test P > 0.05).


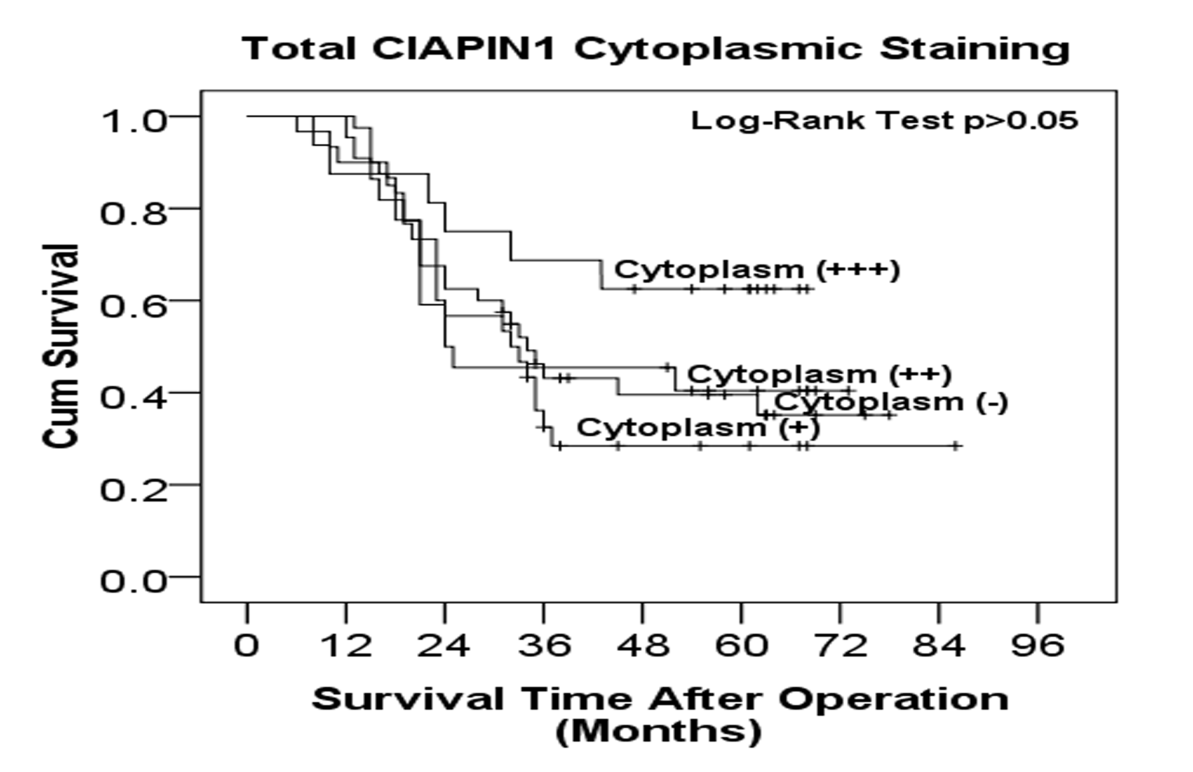

Supplement: Additional file 1 — Table S1. Correlation between CIAPIN1 cytoplasm expression and clinical pathological parameters ( P < 0.05 statistically significant). Figure S1. Kaplan-Meier curves of postoperative survival of patients with cytoplasmic localization of CIAPIN1 expression. Patients with high cytoplasmic expression of CIAPIN1 in EOC; the survival was not statistically significant (log-rank test, P > 0.05). [file 1477-7819-10-112-S1.doc]
